# Extensive Thrombosis Following Lead Extraction: Further Justification for Routine Post-operative Anticoagulation

**DOI:** 10.1016/s0972-6292(16)30755-0

**Published:** 2014-05-25

**Authors:** Mikael Hanninen, Romain Cassagneau, Jaimie Manlucu, Raymond Yee

**Affiliations:** London Health Sciences Centre, Canada

**Keywords:** Lead Extraction, Thrombosis, Post-operative Anticoagulation

## Abstract

Lead extraction is becoming increasingly common as indications for pacing and ICD insertion expand. Periop management varies between extraction centers, and no clinical guidelines have addressed the need for perioperative anticoagulation. We report a case of massive thrombosis which occurred shortly after laser lead extraction and is undoubtedly related to the trauma of the extraction and ensuing hypercoagulabiilty. Routine post-operative anticoagulation has been advocated as a means to prevent access vein (subclavian) stenosis, but many centres do not employ a routine post-extraction anticoagulation strategy. Pulmonary embolism following lead extraction is a known complication of this procedure and late mortality following lead extraction is a significant and underappreciated problem. We propose that further research attention should be directed at addressing the issue of routine post-extraction anticoagulation.

## Case Report

**Figure 1:**
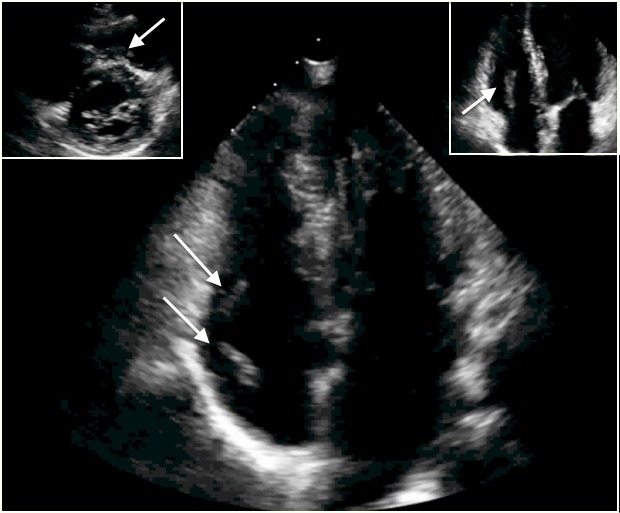
Massive thrombosis noted in the right atrium, right ventricle and right ventricular outflow tract following an otherwise uneventful lead extraction in a patient with a pocket infection

A 60 year-old man was referred to our center for lead extraction after developing a pocket infection following a routine generator change. His original dual-chamber device was implanted in 2002 for symptomatic bradycardia and standard right atrial appendage and right ventricular apical lead positions were confirmed on preoperative chest x-ray. A transesophageal echocardiogram was done to rule out any vegetations or thrombus on the leads and blood cultures drawn before the extraction were negative. The patient was not pacemaker dependent and underwent removal of his pulse generator prior to arriving to our center; swabs from the pocket done at this time showed methicillin-sensitive Staphylococcus aureus. Laser lead extraction was acutely successful and no immediate complications were noted. Post-operative anticoagulation was not administered. A routine transthoracic echocardiogram performed 30 hours following the procedure demonstrated extensive thrombosis extending from the right atrium all the way to the right ventricular outflow (Figure 1). Anticoagulation was immediately started and serial echocardiograms over the ensuing weeks demonstrated complete thrombus resolution.

## Discussion

Lead extraction has become increasingly important as more patients receive cardiac implantable electronic devices (CIEDs). Perioperative management protocols vary widely between centers, including concerning postoperative anticoagulation. The risk of postoperative subclavian vein occlusion, which occurs in at least 8% of cases [1], has been used to justify routine post-operative anticoagulation in certain centres, including ours. However, many centers do not currently employ a strategy of routine postoperative anticoagulation and recent consensus statements concerning lead extraction do not discuss postoperative anticoagulation [2]. Although procedure-related deaths in experienced centers are less than 1%, 30-day and 3-month mortality rates of 2.1% and 4.2% respectively have been reported suggesting room for improvement in postoperative care. The substantial amount of thrombus noted throughout the right-sided cardiac chambers in our case is a particularly impressive example of how the trauma of lead extraction can contribute to massive thrombosis. Pulmonary embolism is a known cause of late death in these patients and in our opinion, this case provides further justification for routine anticoagulation following lead extraction. We propose that the merits of this approach should be studied further in a multicenter, randomized clinical trial.

## References

[R1] Bracke FA (2003). Symptomatic occlusion of the access vein after pacemaker or ICD lead extraction. Heart.

[R2] Wilkoff BL (2009). Transvenous Lead Extraction: Heart Rhythm Society Expert Consensus on Facilities, Training, Indications and Patient Management. Heart Rhythm.

